# Using a Multi-Trait Approach to Manipulate Plant Functional Diversity in a Biodiversity-Ecosystem Function Experiment

**DOI:** 10.1371/journal.pone.0099065

**Published:** 2014-06-04

**Authors:** Conrad Schittko, Mahmoud Hawa, Susanne Wurst

**Affiliations:** Freie Universität Berlin, Functional Biodiversity, Dahlem Center of Plant Sciences, Berlin, Germany; University of Oxford, United Kingdom

## Abstract

A frequent pattern emerging from biodiversity-ecosystem function studies is that functional group richness enhances ecosystem functions such as primary productivity. However, the manipulation of functional group richness goes along with major disadvantages like the transformation of functional trait data into categories or the exclusion of functional differences between organisms in the same group. In a mesocosm study we manipulated plant functional diversity based on the multi-trait Functional Diversity (FD)-approach of Petchey and Gaston by using database data of seven functional traits and information on the origin of the species in terms of being native or exotic. Along a gradient ranging from low to high FD we planted 40 randomly selected eight-species mixtures under controlled conditions. We found a significant positive linear correlation of FD with aboveground productivity and a negative correlation with invasibility of the plant communities. Based on community-weighted mean calculations for each functional trait, we figured out that the traits N-fixation and species origin, i.e. being native or exotic, played the most important role for community productivity. Our results suggest that the identification of the impact of functional trait diversity and the relative contributions of relevant traits is essential for a mechanistic understanding of the role of biodiversity for ecosystem functions such as aboveground biomass production and resistance against invasion.

## Introduction

Biodiversity is declining rapidly [1], which may negatively affect ecosystem functions [2] and economically important ecosystem services at the same time [3]. The role of plant functional diversity on ecosystem properties has been a research focus in recent years [4]–[6]. Several different experiments have shown that positive diversity effects on plant community biomass production mostly result from niche complementarity in combination with a species-specific sampling effect [7]–[10]. The number of functional types or groups (e.g. C_4_ grasses, C_3_ grasses, N-fixing plants, and non-N-fixing plants) represented by the species in a local community is a common measure of functional diversity that is often used to manipulate functional diversity in experimental set-ups [4]–[6], [8]. However, the manipulation of functional group richness in biodiversity-ecosystem function experiments goes along with major disadvantages like the exclusion of functional differences that occur between organisms in the same group [11]. Further, the assumption that ecosystem processes are governed by the abundance and distribution of functional traits in a community [5], [12], [13] has attracted growing attention on the use of functional trait composition, rather than species or functional group richness, in the explanation of biodiversity-ecosystem functioning relationships.

The effects of functional traits on ecosystem properties are quantified by two conceptually different approaches. On the one hand, community-weighted means of trait values (CWM) are commonly computed as mean trait values weighted by the species relative abundances in a given community [14], [15]. This single trait index is interpreted as a trait value expected from a random sample of the community and is theoretically consistent with Grime's [16] mass-ratio hypothesis. On the other hand, a number of new multi-trait approaches such as functional attribute diversity (FAD), functional diversity (FD) or Rao's Q has been introduced which assess functional diversity of a community by quantifying the distances of trait values of species in a multi-dimensional trait space [11], [17]. The concept of functional trait diversity is based on the assumption that greater differences between species' trait values represent greater trait complementarity and larger functional diversity. Since the introduction of the continuous multi-trait Functional Diversity (FD) index by Petchey & Gaston [11], [18] it has been shown that FD explains variation in ecosystem functions, most notably plant aboveground productivity of grassland species [19], [20]. However, multi-trait approaches were solely used for measuring functional diversity either on experimental communities that were compiled by another approach (e.g. species richness or functional group richness) or on natural communities.

So far, no manipulative study has used trait-based functional diversity measures to create different functional diversities during the composition of experimental communities; thus knowledge about their practicability in manipulating functional diversity in experimental set-ups is still missing. Several properties of these measures predict that they will explain greater variance in ecosystem function than either species richness or functional group richness [21]. And, already seven years ago Petchey & Gaston [11] requested experiments that manipulate trait distributions in local assemblages while species richness is held constant, mainly due to their suggested advantage of eliminating the sampling effect.

In the mesocosm study presented here we used the revised version of the FD index by Petchey & Gaston [11] to generate a gradient of functional diversity among plant mixtures while species richness was kept constant. We used a pool of 20 plant species from the urban European flora as our model plant community. Our interest in urban floras is attributed to the fact that cities are remarkably rich in species – usually species richness is greater than in the surrounding areas – and may harbour one of the hot spots of biodiversity [22]. As a novel aspect of this study we treated the attribute of a plant species to be alien or native (hereafter called the ‘floristic status’ after Klotz et al. [23]) as an additional trait that characterizes a species such as life-history or resource capture traits do. We assume that species with different biogeographical histories differ in the number of associations they form with their biotic environment and that this will not only affect the species' own performance but also community-wide ecosystem properties like traditionally used functional traits do. We are aware that the floristic status does not fulfill the definition of a functional trait in its narrowest sense as being any morphological, physiological or phenological plant feature [24]. However, in case of considering a functional trait as a characteristic that is relevant to ecosystem functioning [5], [25], [26] or influences the own performance of an organism [12], the addition of the floristic status as additional trait to the classical functional traits appears reasonable.

In this study we explored firstly whether a positive relationship between functional diversity and aboveground productivity is detectable in plant mixtures that were compiled on the basis of the FD index. This new approach may give insight into the effectiveness and appliance of trait-based approaches for manipulative studies. Secondly, we calculated community-weighted means (CWM) of the selected plant functional traits to determine the relative importance of each trait in explaining the relationship between functional trait diversity and aboveground productivity. In a second phase of the experiment we moved the focus to the resistance to invasion, which is an ecosystem function that was linked with species diversity and functional diversity since the early days of invasion ecology [27]. Experimental studies following a functional approach showed that functional group identity rather than functional group richness limits the invasion process of alien plant species in resident communities [28], [29], which is a finding that is consistent with the sampling effect hypothesis, but in a functional context. In our study, it was the third goal to elucidate the question whether functional trait diversity affects community resistance to invasion. Therefore we added seeds of the exotic Canada goldenrod *Solidago canadensis* to each plant mixture and monitored seed germination and seedling survival.

## Methods

In this study we used a single factor regression design with functional diversity being the single continuous factor that was experimentally manipulated. Functional diversity was calculated for all possible eight-species mixtures from a pool of 20 naturally co-occurring plant species, based on the FD-index introduced by Petchey & Gaston [11], [18]. This measure of functional diversity is defined as the total branch length of a functional dendrogram, which is created from data of plant functional traits. From the total number of possible eight-species mixtures (125,970), 40 mixtures were randomly selected and planted out for the experiment. This design was chosen to cover a broad range of functional diversity (from low FD to high FD).

### Species pool and functional traits

Plant species selection for the species pool of the experiment was conducted with the purpose of reflecting the composition of a ruderal plant community, which is a typical attribute of temporary abandoned fields and disturbed habitats in urban areas. Specific abiotic conditions and the proximity to human settlements and transport connections support the establishments of non-native plant species in these areas [30]. The species pool contained 20 angiosperm species (13 natives and 7 non-natives), of which 15 were forbs, 3 grasses and 2 tree species (see Table S1). The major part of the plant seeds we used was collected on an urban abandoned site in Berlin (N52°28′30", E13°21′46"). This site was open to the public and it was no permission required to enter that site or to collect plant seeds from there. Regarding to the red list of endangered vascular plant species of Berlin [31] none of our experimental plant species were endangered or protected. A minor part of seeds was provided by Appels Wilde Samen (Darmstadt, Germany) or by the Botanical Garden Berlin-Dahlem. To classify each plant species functionally we used data of seven functional traits (four continuous and three categorical) plus the species' floristic status. We aimed to include traits that are related to plant productivity, resource use, coexistence and competitive strength. The categorical traits were: life form, leaf form and resource use strategy (i.e. root colonization with N-fixing bacteria and/or arbuscular mycorrhizal fungi, AMF). The continuous traits were: specific leaf area (SLA), canopy height, seed mass, leaf dry matter content and floristic status. Trait values for each trait were exclusively obtained from trait-databases (BiolFlor [23] and LEDA [32]) or in some cases of unavailability from approved publications. The trait floristic status defines whether a plant is native or alien to Germany. For the specification of the floristic status we followed the classification from the BiolFlor database [23], which is based on the classification system by Schroeder [33]. This classification acts exclusively on a temporal basis excluding information about the evolutionary history, origin or mode of introduction. Alien species are classified according to their time of human introduction: before the discovery of America (archaeophytes, trait value ‘2’ in our data table) and after the discovery of America in 1492 (neophytes, trait value ‘1’). Species that colonized the focus area after the end of the last glacial period (about 12,500 years ago) without human assistance are classified as indigenous or native (trait value ‘3’). Our approach is backed on the aspect that native species have longer evolutionary histories with other local organisms than species that established later in the course of time (see Text S1 for additional information).

### Calculation of Functional Diversity (FD)

The calculations of the FD index for each possible eight-species mixture (125,970 in total) were performed by the R-based software FDiversity [34]. After calculating the FD values we ended up with a range of FD between 1.69 (lowest value) and 4.08 (highest value, see Text S2 for additional information on the calculation). From the 125,970 possible mixtures we randomly chose 40 mixtures with the Excel function ‘randbetween’ (Microsoft Excel 2007).

### Experimental set-up and harvest

In November 2011 we started the experimental part of the study: We used 40 plastic pots (7.5 l volume, 21 cm height, 26 cm upper diameter and 19 cm lower diameter) and filled them with 7 kg soil each. The soil was a loamy sand (pH 8.1, C/N ratio of 28∶1, determined with an elemental analyzer Euro EA, HEKAtech GmbH, Germany) that was collected from an urban abandoned site in Berlin (N52°28′30", E13°21′46") and sieved before the experiment (4 mm mesh size) but remained untreated otherwise. Each of our experimental plant species could be found on that site. 16 equally sized seedlings (two individuals per species) that had been grown on sterilized soil were transplanted carefully into each mesocosm according to the drawn species compositions. Each seedling was assigned by chance to one of 16 possible positions in the mesocosms. The mesocosms were distributed within the greenhouse, watered regularly and their position was randomized twice a week. During the whole experiment the pots were kept in the greenhouse with 16 hours light a day (∼180 µE m-2 sec-1 PAR) and 22/18°C day/night temperature. Nine weeks after transplanting the seedlings into the mesocosms, plant shoots were harvested. Shoots were cut 3.5 cm above ground level to enable regrowth. Shoot weight was determined after drying at 50°C for three days.

### Invasion by *S. canadensis*

To simulate a biological invasion into an established ruderal plant community after a disturbance by cutting we added 100 achenes (hereafter called seeds) of *Solidago canadensis* L. (Asteraceae) evenly to the soil surface of each mesocosm. The Canada goldenrod *S. canadensis* is an invader of North American origin, was introduced in the 18^th^ century in Europe and began to spread in the 19^th^ century in Central Europe where it may become a highly dominant species on abandoned fields and disturbed habitats in urban areas [30]. *S. canadensis* seeds were collected on the same field described above and each seed was checked with a dissecting microscope for its color and size to prevent that they may differ in the ability to germinate. We executed the seed addition after the cutting to prevent that the seeds and seedlings of *S. canadensis* will get strong shading from the established plants. We checked the mesocosms every second day for numbers of newly emerged seeds and survived seedlings until three months after seed addition. Germination and survival rate were calculated per pot.

### Data analysis

Aboveground community biomass at the time of harvest was calculated for each eight-species mixture as the sum of biomass of the 16 individuals per mixture. The influence of FD on aboveground community biomass was tested with a linear regression with FD as the predictor and biomass as response variable. Community-weighted means of trait values (CWM) were calculated for each mixture based on species biomass proportions according to the equation 
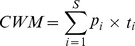
 where S is the number of species in the community, p_i_ is the species biomass proportion and t_i_ are the given trait values. To elucidate the relative contribution of CWMs of traits to the amount of explained variation in aboveground biomass production we performed multiple regressions considering statistical models with different combinations of included explanatory variables (traits). We used the Akaike information criterion (AIC) and *R* ^2^ values to select for the model with the best fit. Afterwards we performed a standardized principle components analysis (PCA) with the CWMs of the traits from the selected model to determine multiple relationships between them [13]. The influence of FD on *S. canadensis* seedling germination was tested with a regression analysis. The regression function of the curve with the best fit to our data was determined by comparing R^2^ and AIC values of different regression functions (linear, exponential, logarithmic, power, polynomial). The percentage of survived seedlings was analyzed with a logistic regression model which is an instance of a generalized linear model. The logistic function avoids predictions below and above 0 and 100% and is widely used to analyze percentage data [35]–[37].

All models were tested for the underlying assumptions (homoscedasticity, normality). All statistical analysis were done in R (version 3.0.1, [38]).

## Results

**Figure 1:**
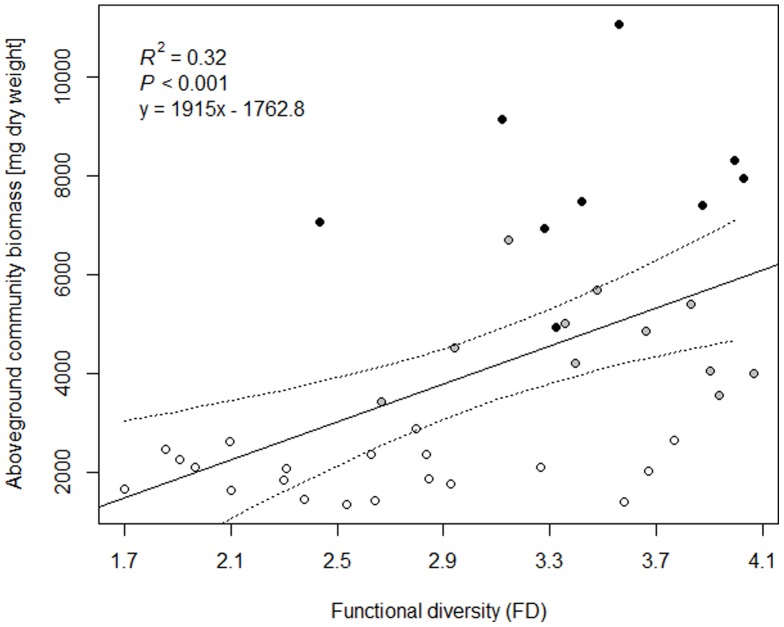
Aboveground community biomass plotted against the FD index of each eight-species mixture. Increasing FD index values represent increasing functional diversity within the mixtures. (intercept  = −1762.8, SE = 1577.7, *t* = −1.1, slope = 1915.0, 95% confidence intervals for slope 889.2–2940.9, SE = 506.8, *t* = 3.8, n = 40). The regression line is solid with 95% confidence bands (dashed lines). Black points represent mixtures that contained the two most productive species of the experiment (*Melilotus albus* and *Medicago* x *varia*). Grey points represent mixtures that contained either *M. albus* or *M*. x *varia*. Empty points represent mixtures that contained neither of these two species.

Statistical analysis of aboveground biomass indicated a positive relationship between FD and biomass production (Fig. 1). Altogether, FD explained 32% of the total variation of biomass among the 40 mesocosms.

The model selection procedure led to a model that included the traits N-fixation, floristic status, AMF and SLA and explained 93% of the variation in aboveground biomass (Table 1). N-fixation, AMF and SLA were positive correlated with aboveground biomass whereas the floristic status showed a negative correlation. In terms of floristic status, high biomass proportions of non-native species in regard to community biomass (hence a low CWM for the trait floristic status) were accompanied with a high community biomass production.

**Table 1:** Summary for the best statistical model based on 4 predictor variables explaining the variation of aboveground community biomass.

Functional trait	*t*-value	*P*-value	Partial *R* ^2^
Nitrogen fixation	11.718	<0.001	positive 0.893
Floristic status	2.115	0.0416	negative 0.337
AMF	1.726	0.0933	positive 0.280
SLA	−1.966	0.0573	positive 0.315
**Degrees of freedom**	35		
**Multiple ** ***R*** **^2^**	0.9304		
***P*** **-value**	<0.001		

**Figure 2:**
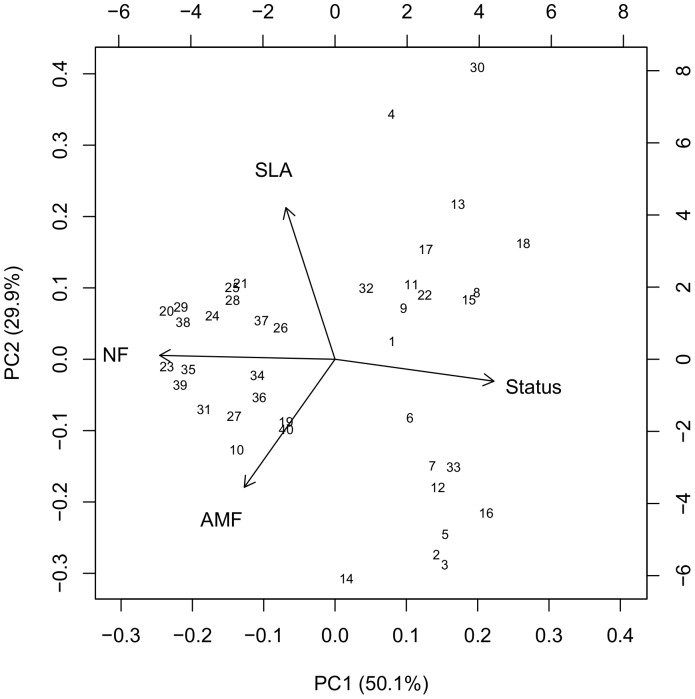
Standardized principal components analysis (PC1 vs. PC2) of the four most important traits. Plotted are the community weighted means (CWM) of the trait values of the functional traits N-fixation (NF), floristic status, specific leaf area (SLA) and arbuscular mycorrhizal fungi (AMF). CWMs of trait values were calculated for each of the 40 mixtures (plotted numbers) based on species biomass proportions.

The two leading axes of a standardized principle component analysis (PCA) on the four selected traits N-fixation, floristic status, SLA and AMF explained about 80% of variation (Fig. 2). The first principal component accounted for 50.1% and had a high positive loading for floristic status as well as a high negative loading for N-fixation. Linear regression between total community aboveground biomass and the first PCA component showed a strong significant negative correlation (*R* ^2^ = 0.75, *P*<0.001) between both variables. The second principal component accounted for 29.9% of variation. SLA had a positive and AMF a negative loading on this axis. Linear regression between total community aboveground biomass and the second PCA component showed no significant correlation (*R* ^2^ = 0.01, *P*>0.05) between both variables.

### Invasion of *S. canadensis*

**Figure 3:**
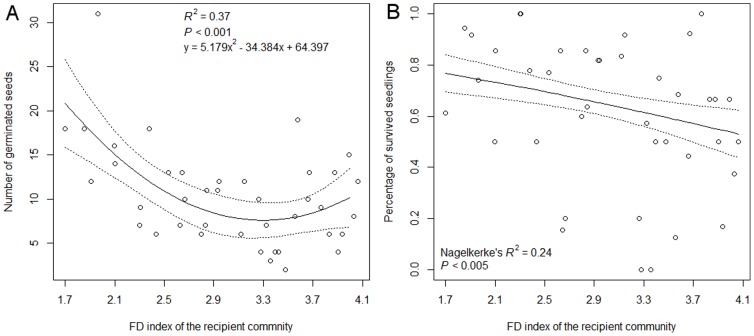
Invasion of *S.canadensis*. (A) Number of germinated seeds of *S. canadensis* from 100 sown seeds in each mesocosm (linear regression, intercept  = 64.4, SE = 14.4, *t* = 4.5, linear coefficient  = −34.4, SE = 10.1, *t* = −3.4, quadratic coefficient  = 5.2, SE = 1.7, *t* = 3.1, n = 40) and (B) the percentage of survived seedlings plotted against the increasing FD index (logistic regression, intercept  = 1.9, SE = 0.4, *z* = 4.5, slope  = −0.5, SE = 0.1, *z* = −3.2, n = 40, note that Nagelkerke's *R* ^2^ is a pseudo *R* ^2^, comparison to standard *R* ^2^ from ordinary least square regression is not appropriate). Regression lines are solid with 95% confidence bands (dashed lines).

Seedling germination rate of *S. canadensis* in the 40 mixtures is best explained by a polynomial function (Fig. 3A). Between FD values 1.7 (lowest FD) and 3.5 the number of germinated seeds decreased with increasing FD value. From 3.5 to 4.1 (highest FD) the number of germinated seeds increased again, but less steep and without reaching germination rates from the lowest FD values. The seedling survival rate showed a negative linear relationship with increasing FD value (Fig. 3B).

## Discussion

A few approaches and tools have been developed for measuring and assessing community's functional diversity based on functional traits which led to our first research question, whether the proposed positive relationship between functional diversity and aboveground productivity is also prevalent when communities were assembled on the basis of the FD index by Petchey & Gaston [11], [18]. A strength of our experimental approach is that species richness is held constant which may lead to the result that a positive functional diversity-productivity relationship would be explained by species' complementarity only, whereas the sampling effect would have been eliminated, as suggested by Petchey & Gaston [11].

### Biomass production is related to functional trait diversity

Our finding that aboveground biomass production is positively related to functional diversity is consistent with the well-known experimental grassland studies like the one carried out at the Cedar creek ecosystem science reserve [4], in California [8], the BIODEPTH sites in Europe [9] or the Jena experiment [10]. By comparing the amount of variation in aboveground biomass production between our small-scale and short-term greenhouse study and these large-scale and long-term field experiments it is notable that functional diversity explained a higher amount of variation in our experiment (32%) than in the other studies (14% at Cedar Creek, 13% at the California experiment, and 15% at the Jena experiment; there is no comparable *R* ^2^ value reported from the BIODEPTH experiment), even though Tilman et al. [39] showed that diversity effects strengthen through time. Spaekova & Leps [40] who manipulated plant species richness in a greenhouse pot experiment also found more pronounced effects of diversity in the greenhouse compared to similar field experiments.

**Figure 4:**
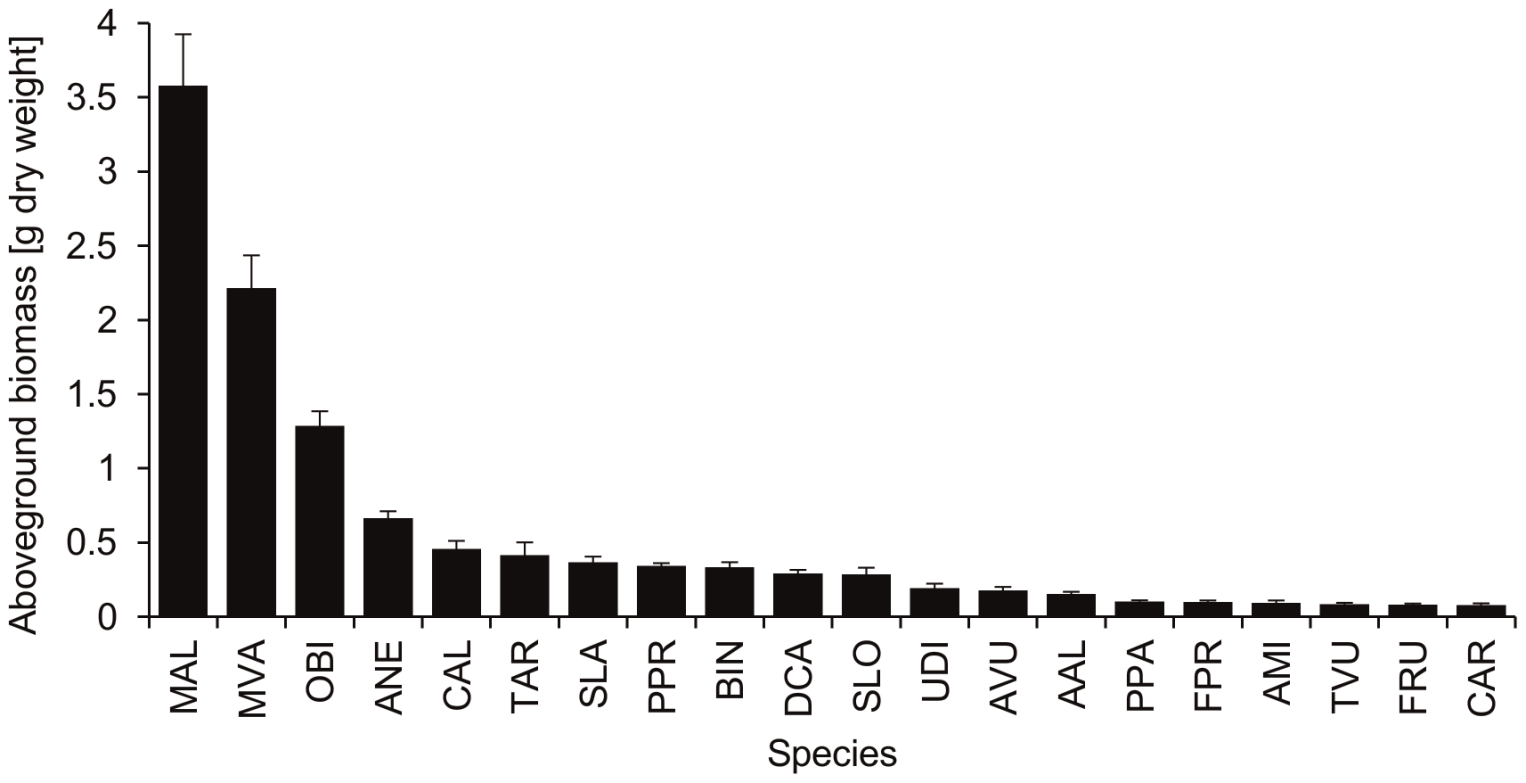
Species-specific plant productivities. Bars represent the mean dry weight of each species from the experiment. Error bars represent SE. Species abbreviations: ANE = *Acer negundo*, AMI = *Achillea millefolium*, AAL = *Ailanthus altissima*, AVU = *Artemisia vulgaris*, BIN = *Berteroa incana*, CAL = *Chenopodium album*, CAR = *Cirsium arvense*, DCA = *Daucus carota*, FPR = *Festuca pratensis*, FRU = *Festuca rubra*, MVA = *Medicago* x *varia*, MAL = *Melilotus albus*, OBI = *Oenothera biennis*, PPR = *Petrorhagia prolifera*, PPA = *Poa pratensis*, SLA = *Silene latifolia*, SLO = *Sisymbrium loeselii*, TVU = *Tanacetum vulgare*, TAR = *Trifolium arvense*, UDI = *Urtica dioica*.

However, the biomass production of the 20 plant species was very uneven in our experiment (Fig. 4). Our most productive species (*Melilotus albus*) was on average over 45 times more productive than our least productive species (*Cirsium arvense*). That the two most productive species (*M. albus* and *Medicago* x *varia*) were both legumes is most likely caused by the N-poor soil we used (which had a C/N ratio of 28∶1). Although we did not manipulate the composition of the randomly chosen mixtures that were planted out for the experiment, the two most productive species were not distributed evenly over the range of the FD index values. Both species entered the experiment at a FD index value at 2.4 which is the point where the community biomass of mixtures that contained one or both of the two species is markedly increased (Fig. 1, black and grey points). Combining this random compositional effect with our finding that the trait N-fixation played the most important role in explaining aboveground productivity leads to the indication that a sampling effect contributed to the positive relationship between functional diversity and productivity in our experiment. The sampling effect is generally defined as an increased probability of including a species of greatest inherent productivity in a plot that is more diverse. That the sampling effect may not only be important at the species level, but also at the trait level, confirms the suggestion of Lepš [41] that life history strategies of constituent species are more important for ecosystem function than species richness. It is also consistent with a recently conducted trait analysis of the Jena experiment by Roscher et al. [13], where the authors showed that increasing community biomass production was best explained by CWMs and suggested that traits of the dominant species were most important for high productivity. In our case, the differences between high productive and less productive species had such a huge dimension that it becomes obvious that the inherent traits of the dominant species are most influential on community productivity when the traits are separately analyzed via the CWM method. Further, we conducted two separated linear regressions, one that included the mixtures without legumes and a second one with all legume-mixtures (data not shown), and found no relationship between FD and productivity (*P*>0.05) within these subsamples, which is another hint that there may have been a sampling effect present in our experiment. However, having a sampling effect in our set-up does not necessarily mean that there was no complementarity among species present. One common form of complementarity in plant communities (which involves both resource partitioning and facilitation) develops between legumes, which have the ability to fix atmospheric N, and other plants, which have access only to soil nitrogen [42]. The sum of both complementarity and sampling effect is the net biodiversity effect [42], and furthermore it is assumed that both may interact with each other [43]. However, within our set-up, it is not possible to identify their individual contributions to the net biodiversity effect since we had no monocultures in our set-up and cannot apply an additive partitioning method.

Data analysis via CWMs also revealed that the species' floristic status was the second most important trait after N-fixation. In our study the loadings of the traits N-fixation and floristic status are opposite in direction on the first PCA axis which tells that the two CWMs have opposite effects, i.e. N-fixation is positively and the floristic status negatively related to productivity. In the case of floristic status it is unlikely that this effect is driven exclusively by a few high productive species, since the three most productive species of our experiment represent all three trait characteristics (native, archaeophyte and neophyte). Moreover, this result is consistent with the finding of a meta-analysis by Vilà et al. [44] that investigated the ecological impacts of exotic plant species and found that exotics have a general negative impact on native species' productivity, abundance and diversity, but they increased total community production by 56.8%.

Our results also showed that not only weighted means of N-fixing plants and exotic species, but also CWMs of a larger set of traits (including the traits SLA and AMF), explained variation in community biomass. But given the facts that *P*-values for both traits are not significant and the second PCA component (on which both traits have high loadings) is not significantly correlated with community biomass indicates that their influence in explaining variation in community biomass is rather negligible [45]. Concerning SLA, this result is consistent with findings from the Jena experiment [13] where the CWM of SLA also did not contribute to the explanation of aboveground productivity.

### Invasion of *S. canadensis*

In terms of seedling survival rate of the invading species *S. canadensis* our results are consistent with previous invasion experiments at small scales, which have shown that increased functional group diversity decreased invasion [46]. The invasion resistance effect has been attributed to the ability of diverse communities to occupy more space, generate more biomass, and use more resources which are than not available to the invading species [39], [47]. But also the trait characteristics of resident species seem to play an important role in the establishment of invading species, i.e. negative and positive effects on the invaders have been associated with specific functional traits of single species within the resident community [5]. For example, strong negative effects on invaders are reported from C_4_ grasses [48], whereas legumes are generally known to have positive effects on invaders [49]. Especially the latter finding is inconsistent with the results from our experiment. We found in the first phase of our study that the dominance of the two productive legumes was highest at intermediate levels of FD, which indicates, that their strong effect on overall community productivity has been responsible for the minimum of the *S. canadensis* seed germination rate at an intermediate FD level. The beneficial role mediated by N-supply, which legumes may have for the invaders in long-term studies and under natural conditions, seems to be not relevant in our short-term study. Maybe this negative short term effect of legumes on invaders also explains why our focal plant community has a low proportion of exotic plant species and a high proportion of legumes at a very early successional stage (i.e. in the first year after abandonment) in the field (Schittko, personal observation). It is also known that alfalfa (*Medicago sativa*, which is one of the parent species of *M.* x *varia*) produces root exudates with strong allelopathic agents that may reduce seed germination of other species by 50% [50]. Allelopathic effects and the strong dominance of legumes at intermediate FD levels may have led to the minimum of the *S. canadensis* seed germination. That the germination rate increased again slightly at high FD levels may be explained by the fact that legume dominance and thereby community biomass was decreased at these levels.

## Conclusions

The approach of using a multi-trait measure for the manipulation of functional diversity can be the superior method because it may explain greater variance in ecosystem functioning than species richness or functional group richness. Our results emphasize the need to incorporate different aspects of functional composition (functional traits, functional diversity) in studies of biodiversity-ecosystem functioning relationships. Besides N-fixation, it was the species' floristic status that explained variation in community productivity second most in our experiment. This finding underlines the fact that exotic plants pose significant impacts at the community and ecosystem level.

In addition, we hope that this article serves as a methodological reference for future ecological multi-species studies in two ways. First, manipulative studies on functional diversity may assume the use of a continuous trait-based approach, and second, the species' attribute of being exotic or native may be seen in a functional context and included in the set of functional traits that are commonly used. In this paper we avoided to label the floristic status as ‘functional trait’ because it breaks with the strict definition of a functional trait as being any morphological, physiological or phenological feature. It should be considered rather as an ‘effect trait’ that can determine effects of plants on ecosystem functions [51]. However, the difficulty in classifying this trait should not prevent community ecologists from its inclusion in studies using trait based approaches, since it can significantly explain variation in functions such as productivity and invasibility.

## Supporting Information

Table S1 **List of the plant species pool and the functional traits.**
(DOC)

Text S1 **Additional information on trait data.**
(DOC)

Text S2 **Additional information on the calculation of FD.**
(DOC)
