# Supplementary material for: Using a Multi-Trait Approach to Manipulate Plant Functional Diversity in a Biodiversity-Ecosystem Function Experiment
Source: PLoS One. 2014 Jun 4;9(6):e99065. doi: 10.1371/journal.pone.0099065 (PMC4045913; doi:10.1371/journal.pone.0099065)
Supplement: Table S1 — List of the plant species pool and the functional traits. (DOC) [file pone.0099065.s001.doc]

**Table S1. List of the plant species pool and the functional traits.**

| **Plant species** | **Family** | **FS** | **LiF** | **RUS** | **SM** | **CH** | **SLA** | **LDMC** | **LeF** |
| --- | --- | --- | --- | --- | --- | --- | --- | --- | --- |
| *Acer negundo* L. | Sapindaceae | 1 | M | AMF | 41.2 | 11.5 | 15.27 | 390 | pinnate |
| *Achillea millefolium* L. | Asteraceae | 3 | H | AMF | 0.1 | 0.5 | 17.32 | 172.2 | pinnatified |
| *Ailanthus altissima* (Mill.) Swingle | Simaroubaceae | 1 | M | AMF | 33.6 | 25.5 | 17.8 | n.a. | pinnate |
| *Artemisia vulgaris* L. | Asteraceae | 3 | H | AMF | 0.1 | 1.4 | 24.122 | 258 | pinnatified |
| *Berteroa incana* (L.) DC. | Brassicaceae | 1 | H | no | 0.7 | 0.3 | 20.62 | 245.24 | long-leaf |
| *Chenopodium album* L. | Amaranthaceae | 2 | T | no | 1.1 | 0.8 | 34.84 | 164.33 | simple |
| *Cirsium arvense* (L.) Scop. | Asteraceae | 3 | G | AMF | 1.2 | 0.9 | 10.07 | 162.1 | pinnatified |
| *Daucus carota* L. | Apiaceae | 3 | H | AMF | 1.1 | 1 | 21.7 | 209.51 | bipinnate |
| *Festuca pratensis s. I.* HUDS | Poaceae | 3 | H | AMF | 2.1 | 0.6 | 33.71 | 279.05 | grass-like |
| *Festuca rubra* L. | Poaceae | 3 | H | AMF | 0.8 | 0.5 | 16.23 | 277.66 | grass-like |
| *Medicago* x *varia* MARTYN | Fabaceae | 1 | C | AMF/NF | 1.75 | 0.4 | 18.1 | 218.66 | palmate |
| *Melilotus albus* MEDIK. | Fabaceae | 2 | H | AMF/NF | 1.9 | 0.9 | 25.37 | 159.25 | palmate |
| *Oenothera biennis* L. | Onagraceae | 3 | H | AMF | 0.4 | 1.1 | 13.5 | 180 | long-leaf |
| *Petrorhagia prolifera* (L.) P.W. Ball et Heywood | Caryophyllaceae | 3 | T | no | 0.3 | 0.3 | 17.63 | 231.22 | long-leaf |
| *Poa pratensis* L. | Poaceae | 3 | H | AMF | 0.3 | 0.3 | 24.57 | 282.15 | grass-like |
| *Silene latifolia* MILL. | Caryophyllaceae | 3 | H | no | 0.8 | 0.6 | 21.625 | 142.5 | simple |
| *Sisymbrium loeselii* L. | Brassicaceae | 1 | H | no | 0.1 | 0.6 | 14.26 | 201.24 | pinnatified |
| *Tanacetum vulgare* L. | Asteraceae | 3 | H | AMF | 0.1 | 0.8 | 16.02 | 221 | pinnatified |
| *Trifolium arvense* L. | Fabaceae | 3 | T | AMF/NF | 0.4 | 0.2 | 20.33 | 317.34 | palmate |
| *Urtica dioica* L. | Urticaceae | 3 | H | AMF | 0.15 | 1.1 | 31.58 | 212.5 | full |

**Abbreviations:** FS=Floristic status (1=Neophyte, 2=Archeophyte, 3=Native), LiF=Life form (M=Macrophanerophyte, H=Hemicryptophyte,
T=Therophyte, G=Geophyte, C=Chamaephyte), RUS=Resource use strategy (NF=Nitrogen fixation, AMF=Arbuscular mycorrhizal fungi),
 SM=Seed mass, CH=Canopy height, SLA=Specific leaf area, LDMC=Leaf dry matter content, LeF=Leaf form.
