# Supplementary material for: Using a Multi-Trait Approach to Manipulate Plant Functional Diversity in a Biodiversity-Ecosystem Function Experiment
Source: PLoS One. 2014 Jun 4;9(6):e99065. doi: 10.1371/journal.pone.0099065 (PMC4045913; doi:10.1371/journal.pone.0099065)
Supplement: Text S1 — Additional information on trait data. (DOC) [file pone.0099065.s002.doc]

**Text S1. Additional information on trait data.**

Values for floristic status, seed mass, life form and leaf form were obtained from the BiolFlor database [1], and values for specific leaf area and leaf dry matter content were taken from the LEDA database [2]. Regarding the LEDA database, we chose the aggregated values from the species fact sheets, and if not available, we used the implemented web query tool to aggregate suitable data manually.

For information about plant association with mycorrhizal fungi we checked appropriate publications [3–7]. Because none of our selected plant species is associated with other mycorrhizal types than arbuscular mycorrhizal fungi (e.g. ectomycorrhizal fungi), there was no other trait values than “no”, “AMF” or “AMF/NF” (the latter means that the plant species is associated with AMF and nitrogen fixing bacteria simultaneously).

The trait floristic status defines whether a plant is native or alien to Germany. For the specification of the floristic status we followed the classification from the BiolFlor database [1], which is based on the classification system by Schroeder [8] and is also used by other authors [9]. This classification acts exclusively on a temporal basis excluding information about the evolutionary history, origin or mode of introduction. Alien species are classified according to their time of human introduction: before the discovery of America (archaeophytes) and after the discovery of America in 1492 (neophytes). Species that colonized the focus area after the end of the last glacial period (about 12,500 years ago) without human assistance are classified as indigenous or native. Contrary to other studies engaged with biological invasions that classify species into the two categories ‘native’ or ‘alien’, we treated the floristic status as a continuous variable based on time of introduction or appearance. This approach is backed on the aspect that native species have longer evolutionary histories with other local organisms than species that established later in the course of time. In our trait matrix, native species were characterized by the trait value ‘3’ (longest history), archeophytes by a value of ‘2’ and neophytes by the value ‘1’ (shortest history). We are aware that the distances between the three trait values are equal but the time ranges that define the categories are unequal. However, having a true continuous variable for the time of species’ appearances would have required accurate data for the evolution and historical distribution of each species; this data were only available for a few species of our pool. Due to this lack we decided to use this three group classification approach (natives, archeophytes, neophytes) which is most commonly used in Central Europe [10] and has data available for all our species.

**Literature cited in Text S1**

1. Klotz S, Kühn I, Durka W (2002) BIOLFLOR - Eine Datenbank zu biologisch-ökologischen Merkmalen der Gefäßpflanzen in Deutschland. Schriftenreihe für Vegetationskunde 38. Bonn: Bundesamt für Naturschutz.

2. Kleyer M, Bekker RM, Knevel IC, Bakker JP, Thompson K, et al. (2008) The LEDA Traitbase: a database of life-history traits of the Northwest European flora. J Ecol 96: 1266–1274.

3. Harley J, Harley E (1987) A check-list of mycorrhiza in the British flora. New Phytol 105: 1–102.

4. Peat H, Fitter A (2006) The distribution of arbuscular mycorrhizas in the British flora. New Phytol 125: 845–854.

5. Wang B, Qiu Y-L (2006) Phylogenetic distribution and evolution of mycorrhizas in land plants. Mycorrhiza 16: 299–363.

6. Huebner C, McQuattie C, Rebbeck J (2007) Mycorrhizal associations in *Ailanthus altissima* (Simaroubaceae) from forested and non-forested sites. J Torrey Bot Soc 134: 27–33.

7. Bainard L, Klironomos J, Gordon A (2011) The mycorrhizal status and colonization of 26 tree species growing in urban and rural environments. Mycorrhiza 21: 91–96.

8. Schroeder FG (1968) Zur Klassifizierung der Anthropochoren. Veg Acta Geobot 16: 225–238.

9. Kowarik I (2010) Biologische Invasionen. Stuttgart: Ulmer. 320 p.

10. Wittig R (2004) The origin and development of the urban flora of Central Europe. Urban Ecosyst 7: 323–329.
