# Supplementary material for: Using a Multi-Trait Approach to Manipulate Plant Functional Diversity in a Biodiversity-Ecosystem Function Experiment
Source: PLoS One. 2014 Jun 4;9(6):e99065. doi: 10.1371/journal.pone.0099065 (PMC4045913; doi:10.1371/journal.pone.0099065)
Supplement: Text S2 — Additional information on the calculation of FD. (DOC) [file pone.0099065.s003.doc]

**Text S2. Additional information on the calculation of FD.**

In R [1] we used the function combn() in the ‘utils’ package to create a matrix with every possible eight-species mixture from the 20-species pool. With the function melt() in the ‘reshape’ package we transformed that matrix into a table. Further actions were performed by the R-based software FDiversity [2], which is a free tool developed to estimate and analyze indices of functional diversity. Here we merged the species mixtures table with our trait matrix. Each categorical variable was transformed beforehand into several binary variables (also called dummy variables). Afterwards, we calculated the community based FD value of every eight-species mixture (125,970 in total). The community based FD index [3] is a revised version of the plot based version introduced in 2002 [4]. In order to construct the functional dendrogram from which FD is measured one needs to calculate pair wise distances between species, and then use a clustering algorithm. As distance measure we used Gower distance because of its ability to cope with mixed trait data (categorical and continuous) and missing values [5,6]. We used UPGMA (average linkage) as clustering algorithm since Podani & Schmera [6] showed the UPGMA algorithm outperformed other procedures (e.g. UPGMC, WPGMC, single linkage, complete linkage, etc.) in measuring FD indices.

From the 125,970 mixtures we randomly chose 40 mixtures with the Excel function randbetween(). We interfered to the procedure when it happened by chance that FD values of the chosen mixtures resembled each other within two positions behind the decimal point. In that cases we discarded one of the two values and drew a new mixture to achieve an even distribution of FD Values along their full range within the 40 plant mixtures.

**Literature cited in Text S2**

1. R Core Team (2013) R: A language and environment for statistical computing. R Foundation for Statistical Computing, Vienna, Austria.

2. Casanoves F, Pla L, Di Rienzo JA, Díaz S (2011) FDiversity: a software package for the integrated analysis of functional diversity. Methods Ecol Evol 2: 233–237.

3. Petchey OL, Gaston KJ (2006) Functional diversity: back to basics and looking forward. Ecol Lett 9: 741–758.

4. Petchey OL, Gaston KJ (2002) Functional diversity (FD), species richness and community composition. Ecol Lett 5: 402–411.

5. Petchey OL, Gaston KJ (2007) Dendrograms and measuring functional diversity. Oikos 116: 1422–1426.

6. Podani J, Schmera D (2006) On dendrogram-based measures of functional diversity. Oikos 115: 179–185.
